# Transcriptomic Interpretation on Explainable AI-Guided Intuition Uncovers Premonitory Reactions of Disordering Fate in Persimmon Fruit

**DOI:** 10.1093/pcp/pcad050

**Published:** 2023-05-24

**Authors:** Kanae Masuda, Eriko Kuwada, Maria Suzuki, Tetsuya Suzuki, Takeshi Niikawa, Seiichi Uchida, Takashi Akagi

**Affiliations:** Graduate School of Environmental and Life Science, Okayama University, 1-1-1 Tsushimanaka, Kita Ward, Okayama, 700-8530 Japan; Graduate School of Environmental and Life Science, Okayama University, 1-1-1 Tsushimanaka, Kita Ward, Okayama, 700-8530 Japan; Graduate School of Environmental and Life Science, Okayama University, 1-1-1 Tsushimanaka, Kita Ward, Okayama, 700-8530 Japan; Gifu Prefectural Agricultural Technology Center, 729-1 Matamaru Gifu, 501-1152 Japan; Gifu Prefectural Agricultural Technology Center, 729-1 Matamaru Gifu, 501-1152 Japan; Faculty of Information Science and Electrical Engineering, Kyusyu University, 744 Motooka, Nishi Ward, Fukuoka, 819-0395 Japan; Graduate School of Environmental and Life Science, Okayama University, 1-1-1 Tsushimanaka, Kita Ward, Okayama, 700-8530 Japan; Japan Science and Technology Agency (JST), PRESTO, 4-1-8 Honcho, Kawaguchi, Saitama, 332-0012 Japan

**Keywords:** Artificial intelligence, Backpropagation, Convolutional neural network, Image diagnosis, Physiological disorder

## Abstract

Deep neural network (DNN) techniques, as an advanced machine learning framework, have allowed various image diagnoses in plants, which often achieve better prediction performance than human experts in each specific field. Notwithstanding, in plant biology, the application of DNNs is still mostly limited to rapid and effective phenotyping. The recent development of explainable CNN frameworks has allowed visualization of the features in the prediction by a convolutional neural network (CNN), which potentially contributes to the understanding of physiological mechanisms in objective phenotypes. In this study, we propose an integration of explainable CNN and transcriptomic approach to make a physiological interpretation of a fruit internal disorder in persimmon, rapid over-softening. We constructed CNN models to accurately predict the fate to be rapid softening in persimmon cv. Soshu, only with photo images. The explainable CNNs, such as Gradient-weighted Class Activation Mapping (Grad-Class Activation Mapping (CAM)) and guided Grad-CAM, visualized specific featured regions relevant to the prediction of rapid softening, which would correspond to the premonitory symptoms in a fruit. Transcriptomic analyses to compare the featured regions of the predicted rapid-softening and control fruits suggested that rapid softening is triggered by precocious ethylene signal–dependent cell wall modification, despite exhibiting no direct phenotypic changes. Further transcriptomic comparison between the featured and non-featured regions in the predicted rapid-softening fruit suggested that premonitory symptoms reflected hypoxia and the related stress signals finally to induce ethylene signals. These results would provide a good example for the collaboration of image analysis and omics approaches in plant physiology, which uncovered a novel aspect of fruit premonitory reactions in the rapid-softening fate.

## Introduction

Non-invasive prediction of the fate to be disordering is a big issue for plant phenotyping, both in plant biology and agriculture. Genetic mutants would follow a uniform fate, whereas physiological disorders in natura (especially in crops) often occur randomly at one glance, dependent on complicated environmental conditions. Expert skills with long experiences in each specific field would often allow the high-quality prediction to capture the early symptoms, which might enable the characterization of early physiological processes causing the objective disorder. Although acquiring such skills would require a long time and special environments, recent progress in machine learning techniques may allow the reproduction of a professional eye on a specific internal disorder.

Recent progress in machine learning frameworks, such as deep neural networks (DNNs), has realized various image analyses or natural language processing. Especially for image analyses, convolutional neural networks (CNNs) (LeCun et al. 2015) outperformed the conventional machine learning models in the ImageNet Large Scale Visual Recognition Challenge, which tries classification of 1,000 visual object categories (Krizhevsky et al. 2017, Russakovsky et al. 2015). In plant science, CNN techniques have also been applied to various tasks using plant images. For example, a review by Masuda and Akagi (2022) lists the following tasks: taxonomic classification, stress/disease diagnosis, non-invasive prediction, regression and quantification, and automated sorting.

One of the practical issues with DNNs for biology is their ‘black-box’ nature, which prevents us from knowing the reason for their prediction/diagnosis. Consequently, we could not localize the regions contributing to the analysis results. However, the recent development of visualization techniques called ‘explainable AI (X-AI)’ has solved this critical issue. Representative X-AI techniques are Gradient-weighted Class Activation Mapping (Grad-CAM) (Selvaraju et al. 2016), guided backpropagation (Springenberg et al. 2015), guided Grad-CAM (Selvaraju et al. 2017) and layer-wise relevance propagation (Bach et al. 2015).

X-AI techniques may provide valuable interpretations that researchers have not been able to describe. For example, a combination of CNN and X-AI techniques successfully visualizes the early symptoms of disease infection or stress exposure (Ghosal et al. 2018, Nagasubramanian et al. 2020). X-AI has also been applied to reveal invisible feature characteristics of fruit internal traits, such as calyx-end cracking disorder (Akagi et al. 2020) and seedlessness (Masuda et al. 2021) in persimmon fruits. Integrating phenotypic collinearity and feature visualization with X-AI in citrus fruit also realizes generalized interpretations for fruit peelability and hardness (Minamikawa et al. 2022). These studies suggest that X-AI can immediately reproduce professional eyes to describe the contributing region for predicting a specific objective (or phenotype). This allows cell- or region-specific analyses of the biological index for objective phenomena. Hence, the combination of X-AI and plant omics approaches will provide a novel aspect for assessing various physiological reactions in plants.

**Fig. 1:**
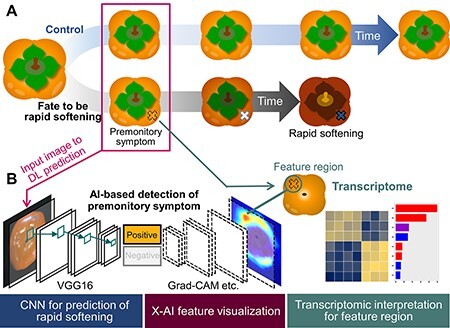
A schematic view of the approach in this study. (A) Fates of persimmon fruits to be rapid softening or long shelf life (control). At harvest, fruits that are rapidly softening are hard to detect even by human experts, but deep learning frameworks can predict them from the faint premonitory symptoms in photo image data (Suzuki et al. 2022). (B) Deep learning training, feature visualization and biological interpretation steps in this study. A VGG16 model is trained with the photo image data of persimmon fruits to classify into rapid softening (positive) and control (negative). The trained model predicts the fates of new testing samples, followed by visualization of the feature region, or potential premonitory symptom, with X-AI techniques, such as Grad-CAM. The feature regions are subjected to transcriptomic analysis to interpret the physiological reactions that occurred there.

Persimmon is a major fruit crop, especially in East Asia. Their fruit disorders, including calyx-end cracking or rapid (over-)softening (or rapid fruit decaying with severe water–soaked patches), substantially involve their commodity qualities, while the physiological mechanisms for their disorder occurrences have been little known (Yamada et al. 1988, 2002). Particularly rapid softening is becoming a serious issue, potentially not only in persimmon fruit. This disorder randomly occurs within ca. 10 d after the harvest even in identical shelf conditions. Although it is so hard to predict rapid softening from outer appearances at harvest even by experts’ eyes, our previous study has developed CNN-based prediction models, only with simple Red, Green, Blue (RGB) photo images (Suzuki et al. 2022). Importantly, the application of an X-AI technique, Grad-CAM, suggested that a few specific regions in the fruit surface, perhaps corresponding to the premonitory symptoms, had substantial weight for the prediction of the rapid-softening fate. This situation might propose a good experimental framework to characterize physiological reactions in the premonitory symptoms of rapid softening. Here, we attempted to perform a comparative transcriptomic analysis on the fruits with a predicted fate to be disordered or to be a control (or with a long storage term) (**Fig. 1**). This would contribute not only to the understanding of the physiological mechanism for rapid-softening fruit but also to the development of a novel approach based on a collaboration of image analysis and plant omics.

## Results and Discussion

### Prediction of rapid-softening persimmon fruits with CNN models

A total of 2,690 persimmon fruits from cv. Soshu were harvested at the same full maturing stage (skin color chart = 6), in October 2018, in Gifu city, Japan. Immediately after the harvest, they were divided into two groups each consisting of 1,446 and 1,244 fruits, and RGB photographic images of the apex side were taken with different digital cameras on a uniform black background to form ‘dataset A’ and ‘dataset B’, respectively (see the Materials and Methods section for the detailed conditions). We have mainly two reasons to apply two digital cameras: (i) for efficient image capturing and (ii) for examination of the robustness of the CNN models trained with images including environmental differences. For the assessment of rapid softening, packaged mature fruits were stored at ambient temperature for 1 week to check their flesh texture, according to the criteria previously used (Suzuki et al. 2022) (see the Materials and Methods section for the details). Fruits substantially softened in 1 week after the harvest were defined as ‘rapid softening’. Here, we applied a typical CNN model called VGG16 for binary classification of positive (or rapid softening) and negative (or control). The model was implemented in Keras 2.2.4 (https://keras.io/) and pre-trained with the ImageNet (http://www.image-net.org/), according to the previous study of training CNN models for predicting persimmon rapid softening (Suzuki et al. 2022).

**Fig. 2:**
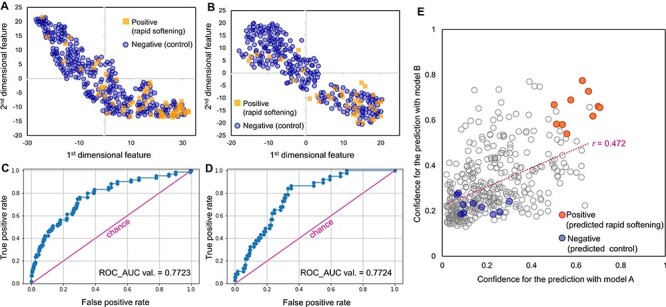
The deep learning prediction of rapid-softening persimmon fruits. (A, B) The distribution of the first and second highest dimensional features in the fully connected layer of the trained VGG16 (model A for (A) and model B for (B)), with the tSNE analysis, which is a dimensionality reduction technique. The squares and circles represent the positive and negative validation samples, respectively (*N* = 361). (C, D) ROC curves for the classification performance of models A (C) and B (D). The line indicates the chance classification. (E) The distribution of the confidence values for the prediction of rapid softening (or positive) in the testing samples, with models A and B. The confidence values for the prediction of 1 and 0 strongly supported rapid softening and control, respectively. The samples highlighted with solid circles were used for the following transcriptomic analyses.

We trained two independent classification models using datasets A and B, respectively. The images in each dataset were randomly split for training and validation sets in a ratio of 3:1. Hereafter, they are called ‘model A’ and ‘model B’ (**Supplementary Fig. S1** shows their training curves). Classification performance was examined by observing the feature distribution in the fully connected layer by t-distributed stochastic neighbor embedding (tSNE), receiver operating characteristic (ROC) curve (**Fig. 2**) and classification accuracies. Both models achieved adequate classification performance [ROC-area under curve (AUC) value > 0.77 and >75% accuracy]. The confidence distributions of these two models were not appreciably correlated [*r* = 0.314 in the validating samples in dataset A (*N* = 361), **Supplementary Fig. S2**]. This suggested that the two models apply slightly different feature characteristics for the classification and that combination of them might achieve higher prediction performance for positive samples (rapid-softening fruits), which could be further analyzed using transcriptomic approaches. The two models, especially model B, exhibited substantially low precision values for the positive classification (positive precisions were 0.47 and 0.25, and negative precisions were 0.89 and 0.97 for models A and B, respectively). For the validation samples in dataset A (*N* = 361), the combination of models A and B, filtering with a higher confidence threshold than in the default classification (=0.5), maximized accuracy > 93% and positive precision > 0.8 (**Supplementary Fig. S2**).

Regarding the selection of test samples for the transcriptomic analysis described below, a total of 311 persimmon fruits of cv. Soshu were harvested in October 2020 in Gifu city, Japan, under the same fruit conditions as in 2018. The trained classification models A and B were applied to predict their fate to soften rapidly (positive) or not (negative). We selected 10 positive samples with the highest confidence by both models A and B, and 10 negative samples with enough low confidence (**Fig. 2E**). It would be worth noting that previous results suggested a substantial correlation between the confidence for the prediction of rapid softening and the actual date to be softened, as rapid softening is a quantitative disorder (Suzuki et al. 2022). Thus, although our selection might include potential false positive samples, they were estimated to be softened more rapidly than the selected negative samples. These 10 positive and negative fruit samples were applied to the transcriptomic analyses described below.

### Visualization of the feature characteristics for the prediction

**Fig. 3:**
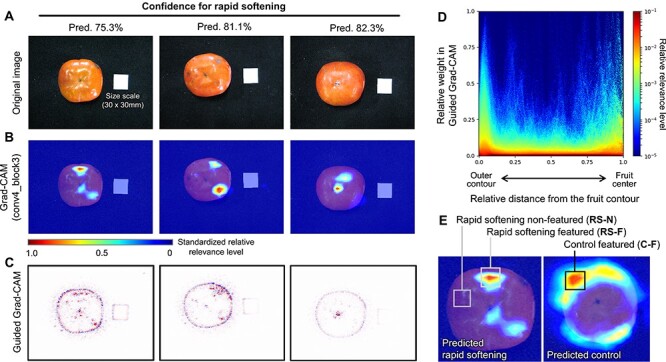
Visualization of the relevant regions for the rapid-softening prediction. (A) Original photo images of the predicted rapid-softening fruit with high confidence. (B, C) Relevance visualization with Grad-CAM (B) and guided Grad-CAM (C). The areas with the highest relevance were highlighted. (D) The distribution of relevance weight in fruits, as detected by guided Grad-CAM. A two-dimensional histogram *H*(*r, d*), where *r* is the relevance weight of a pixel and *d* is the normalized distance of the pixel from the outer contour of the fruit. Relatively higher relevance weights were accumulated around the apex (or fruit center) ($d \in \left[ {0.75,\,1.0} \right]$) or in the peripheral regions (or surrounding outer contour) of fruit ($d \in \left[ {0,\,0.1} \right]$). (E) Three categories of the fruit samples based on the rapid-softening prediction and the featured/non-featured regions were used in the following transcriptomic analyses.

For visualization of the regions relevant to the prediction of rapid softening, Grad-CAM (Selvaraju et al. 2016) and guided Grad-CAM (Selvaraju et al. 2017) were applied according to the previous study (Akagi et al. 2020). We detected the relevant regions in 62 predicted rapid-softening fruits with model A in the testing sample sets. **Fig. 3A****–****C** shows three examples with relatively high confidence for the prediction. Although we had hypothesized that physically damaged regions might exhibit higher relevance, the actual relevance was located randomly at a glance or potentially in the regions with color unevenness, as suggested in the previous study (Suzuki et al. 2022). Grad-CAM tries to find the relevant regions in the feature map by the last convolutional layer (conv5_block3 in VGG16). As the feature map is smaller than the original image, Grad-CAM often gives too coarse relevance visualizations (Selvaraju et al. 2017). To improve this situation for finding the relevance with Grad-CAM, we applied a shallower convolutional layer, conv4_block3 in VGG16, instead of the original last convolutional layer, conv5_block3. The visualized relevance was mostly consistent between Grad-CAM and guided Grad-CAM (**Fig. 3B, C**, **Supplementary Fig. S3**). The distribution of the quantitative relevance as a function of the distance from the outer contour of the fruit was detected with 62 predicted rapid-softening fruits in the testing sample set (**Fig. 3D**). Relatively higher relevance distributes mainly around the apex ($d \in \left[ {0.75,\,1.0} \right]$) or in the peripheral regions of fruit ($d \in \left[ {0,\,0.1} \right]$), where *d* is the normalized distance of the pixel from the fruit contour (**Fig. 3D**). In the empirical knowledge, the indexes of certain stresses in persimmon fruits often appear mainly in color unevenness in the peripheral regions, as with fruit calyx–end cracking (Yamada et al. 1988, Akagi et al. 2020). Although these observations are partially consistent with the empirical prediction of rapid softening, most of the relevant regions, especially around the apex, have not been interpretable at least from the empirical knowledge.

Regarding each 10 predicted rapid-softening (positive) and control (negative) samples selected for the following transcriptomic analysis (**Fig. 1E**), we sampled approx. 10 mm × 10 mm × 5 mm (length × width × thickness of the mesocarp) of mesocarp immediately beneath the high relevance for the prediction of rapid softening (RS-featured or RS-F) and control (C-featured or C-F) and with no relevance in the predicted rapid softening (RS-non-featured or RS-NF) (**Fig. 3E**).

### Transcriptomic interpretation of the deep learning prediction

**Fig. 4:**
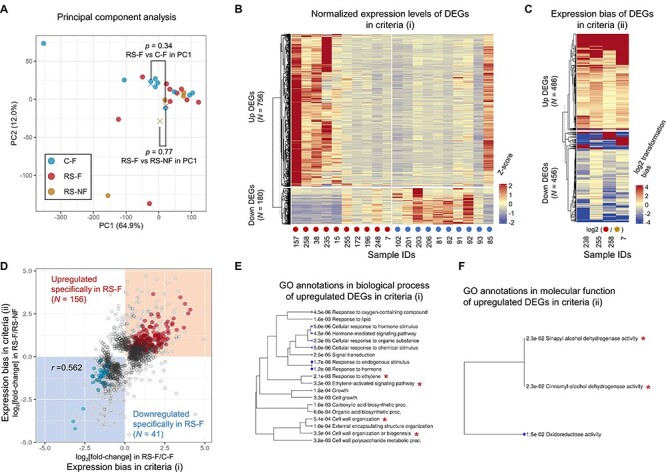
Transcriptomic interpretation of the deep learning prediction on rapid softening in persimmon fruit. (A) The characterization of gene expression dynamics in C-F, RS-F and RS-NF, with PCA. All sample groups were not statistically separated (*P* > 0.34) in PC1 explaining ca. 65% of the whole variance. The cross marks represent the average PC1 and PC2 values in each group. (B, C) The heat map for the normalized expression patterns of DEGs in criteria (i) with C-F and RS-F (B) and criteria (ii) with RS-F and RS-NF (C). (D) The potential correlation of the expression bias in DEGs in criteria (i) (*x*-axis) and criteria (ii) (*y*-axis). The genes commonly upregulated or downregulated in RS-F (*P* < 0.05 and FDR < 0.1 in criteria (i) and (ii), respectively) were subjected to GO enrichment analysis. (E, F) GO enrichment analysis for the upregulated DEGs detected in criteria (i) and (ii) ((E) and (F), respectively, FDR < 0.05). The larger blue dots indicate more significant FDR values. Asterisks represent GO annotations mentioned in the main text.

We obtained mRNA-seq reads from the 10 RS-F, 4 RS-NF and 10 C-F samples. A principal component analysis (PCA) was conducted to profile the expression patterns of all genes [reads per kilobase of transcript per million mapped reads (RPKM) > 1] among the sampling groups (**Fig. 4A**). PC1 and PC2 represented 64.9% and 12.0% of the total variance, respectively. The PCA analysis suggested that the overall gene expression levels have not changed significantly across the sample groups (*P* = 0.34–0.77 in PC1, two-sided Student’s *t*-test). Differentially expression analyses were conducted in the two criteria: (i) RS-F vs C-F and (ii) RS-F vs RS-NF, with DESeq2 and paired edgeR, respectively (**Fig. 4B, C**). The differentially expressed genes (DEGs) in criteria (i) would reflect the difference in the early physiological reactions of fruit with the two distinct ripening fates, rapid softening and long shelf life. On the other hand, criteria (ii) would reflect the physiological reaction in the premonitory symptoms within a rapid-softening fruit. In criteria (i), not all but half of the RS-F samples (left five samples in **Fig. 4B**) exhibited clearly consistent both up- and down regulations in comparison to the control samples. The rest half RS-F samples exhibited no clear upregulations, but consistent tendency for the expression patterns in the downregulated DEGs. This situation would be derived from sample heterogeneity. On the other hand, one of the control samples (ID number 85, right end in **Fig. 4B**) exhibited clear RS-F-like expression patterns. This might be due to potentially wrong prediction of the control sample, since the prediction performance with our model was not perfect (negative precision = ca. 0.95, as given in **Supplementary Fig. S2**). On the other hand, in criteria (ii) with a paired analysis, four comparisons of RS-F and RS-NF exhibited mostly consistent expression behaviors in DEGs (**Fig. 4C**).

We could detect 756 and 180 RS-F upregulated and downregulated DEGs using DESeq2 (*P* < 0.05, RPKM > 1 for either of the averaged value in RS-F or C-F), respectively, in criteria (i). The RS-F upregulated genes were statistically enriched with ethylene signal–related and cell wall modification genes [**Fig. 4E** for the gene ontology (GO) enrichment analysis in the biological process, and **Supplementary Table S1** for the DEG list]. They clearly reflected a typical physiological reaction in softening of climacteric fruit species, such as tomato (Alexander and Grierson 2002), where ethylene plays a main role in eventually inducing cell wall (or polysaccharide) degradation enzymes, including pectin lyase (or pectin methylesterase) (see **Supplementary Table S1** for the details). This result suggested that genes inducing typical fruit softening were precociously activated in the fruits to be rapid softening, although we (and also empirical knowledge) cannot detect clear visible indexes in outer phenotypes between the rapid softening and control persimmon fruits (Suzuki et al. 2022). On the other hand, RS-F-downregulated genes showed significant enrichment of membrane transport–related and immune- or defense-related genes (**Supplementary Fig. S4B**). This is consistent with the reactions associated with the upregulated genes, since fruit cells already in the decaying process have no longer the membrane transporting or defending abilities, as indicated in the reactions with fruit chilling injury (Lyons 1973, Saltveit and Morris 1990).

**Fig. 5:**
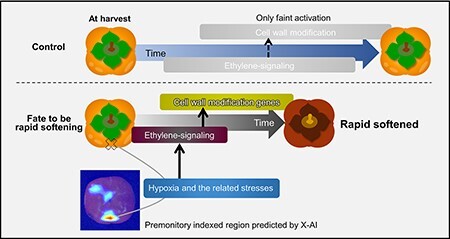
A schematic model for the physiological mechanism of rapid softening in persimmon fruit.

For criteria (ii), we detected 486 and 456 RS-F upregulated and downregulated DEGs using paired edgeR (FDR < 0.1, RPKM > 1 for either of the averaged value in RS-F or RS-NF, and **Supplementary Table S2** for the DEGs list), respectively. Their significance is less interpretable from the significantly enriched gene ontologies in the biological process (**Supplementary Fig. S4C**) than that of criteria (i) (**Fig. 4E**). The RS-F upregulated genes were enriched in those annotated with alcohol dehydrogenase (ADH) in molecular function (**Fig. 4F**), similarly to those upregulated in criteria (i) (**Supplementary Fig. S4A**). This is reminiscent of a typical response to hypoxic conditions (or low oxygen stress) in fruit crops, including avocado, tomato or pear (Kanellis et al. 1991, Van Der Straeten et al. 1991, Chervin et al. 1999). Upregulation of *ADH* has been known as an index of hypoxia also in persimmon, mainly in the process of fruit deastringency with CO_2_ treatment (Min et al. 2012, Zhu et al. 2018). The RS-F upregulated genes also included a few ethylene-related genes, such as *1-aminocyclopropane-1-carboxylate oxidase* or *1-aminocyclopropane-1-carboxylate synthase*, and genes relating cell wall modification, such as *pectin methylesterase*, although they exhibited no drastic activation (approx. 1.3–2.9-fold changes in average, **Supplementary Table S2**) and no significant enrichment (**Fig. 4C**). These observations suggest that partial activation of ethylene-signaling pathways precedes cell wall degradation in the featured region–specific manner. The RS-F-downregulated genes exhibited the enrichment of brassinosteroid- or abscisic acid–responsive genes (**Supplementary Fig. S4D**). This might be consistent with the gene regulation in criteria (i), where defense-responsive abilities have been gradually lost. In the comparison of criteria (i) and (ii), relatively high correlation was detected in their DEG expression behaviors (*r* = 0.562, **Fig. 4D**). Among the DEGs in criteria (i) and (ii), 156 and 41 genes showed the common RS-F–specific upregulation and downregulation, respectively (**Fig. 4D**, and **Supplementary Table S3** for the gene list). Notably, *ADH*-related genes were commonly enriched in the upregulated DEGs of RS-F in both criteria (i) and (ii) (**Supplementary Fig. S4E**), implicating low oxygen stress in persimmon fruit, as described earlier. Together, our transcriptomic results suggested that the Deep Learning-predicted rapid-softening fruits precociously start an ethylene-dependent cell wall degradation process and that the cue to trigger this reaction might involve certain stress signals potentially due to hypoxia, as given in **Fig. 5**. These results would provide a good example for the collaboration of AI and omics approaches in plant physiology, which uncovered a novel aspect of fruit premonitory reactions in the rapid-softening fate.

## Materials and Methods

### Assessment of fruit softening in cv. Soshu persimmon

A total of 2,690 cv. Soshu persimmon fruits were harvested in the Itonuki area Gifu, Japan, at fully mature stage (skin color chart = 6) in October 2018. Immediately after harvest, the RGB images (2,048 × 1,536 pixels) from the fruit apex side were taken at uniform light and background conditions using two digital cameras (Canon IXY-Digital 20IS and Nikon D5200). We defined the fruits over-softened in 7 d after the harvesting during storage at room temperature as positive samples for rapid softening. Fruits that did not return dented after being touched according to Sugiura et al. (2012) and Suzuki et al. (2022) were recorded as softened fruit.

### Deep learning model construction for the prediction of rapid softening

The procedure of deep learning classification was followed by the previous report (Suzuki et al. 2022). Roughly speaking, we prepared an image dataset consisting of the softened fruits in 7 d as positive samples and the long shelf fruits (>7 d for softening) as negative samples for binary classification. All images were resized to 224 × 224 pixels and augmented with the ImageDataGenerator in Keras (https://keras.io/). Then images were randomly split for training and validation sets in a ratio of 3:1. The VGG16 model (Simonyan and Zisserman 2014) was implemented in Keras 2.2.4, and its fully connected layer was customized for binary classification. Its model was weighted with the ImageNet dataset (http://www.image-net.org/) for pretraining. We used the standard setting with stochastic gradient descent as the solver, 0.001 as the learning rate with categorical cross-entropy for the loss function and 20–100 epochs with the class weight option, since its applicability had already been confirmed by Suzuki et al. (2022). The classification performance of the trained models was evaluated with feature distribution in the fully connected layer using the tSNE and ROC-AUC values in the validation samples. For the confusion matrix, the threshold prediction value was set as 0.5.

### X-AI feature visualization in models

Based on previous reports (Akagi et al. 2020, Suzuki et al. 2022), the feature visualization methods Grad-CAM (Selvaraju et al. 2016) and guided Grad-CAM (Selvaraju et al. 2017) were applied to the trained VGG16 model to find the high-relevance regions. As described in the Results and Discussion section, Grad-CAM is often used to capture the relevant regions in the feature map by the last convolutional layer (conv5_block3). On the other hand, since we need to sample small fruit regions for the following transcriptomic analyses (or to visualize the relevance with a finer resolution than the original one), we applied a shallower convolutional layer using conv4_block3 to find the relevant regions. We have checked that the relevance was mostly consistent not only between Grad-CAM and guided Grad-CAM but also between the Grad-CAM using conv4_block3 and the original Grad-CAM (**Supplementary Fig. S3**). The distribution of the quantitative relevance in guided Grad-CAM, along with the distance from the outer contour of the fruit, was calculated according to the previous method (Akagi et al. 2020).

### Transcriptomic analysis

In October 2020, a total of 311 cv. Soshu persimmon fruits were harvested in the Itonuki area, Gifu, Japan, at a fully mature stage (color chart = 6), of which the condition was mostly identical to that of the described sampling for the CNN model construction. Immediately after the harvest, photo images shot by the described two cameras were analyzed with the constructed CNN models to predict their fates to be rapidly softening (positive) or to be controlled (negative) with confidence values. We selected 10 predicted rapid-softening fruits with the highest positive confidence and 10 control samples. Grad-CAM and guided Grad-CAM detected the featured and non-featured areas in the fruits. The fruit exocarps and mesocarps around the highest featured areas [10 mm × 10 mm × 5 mm (length × width × thickness of the mesocarp)] were sampled to be immediately frozen in liquid nitrogen. The remaining fruits were stored at room temperature, and their dates of fruit softening were recorded.

Total RNA was extracted from the frozen samples using PureLink Plant Reagent (Invitrogen, Carlsbad, CA, USA). Illumina sequencing libraries were prepared as previously described (Masuda et al. 2022). Briefly, mRNA was isolated using the Dynabeads^TM^ mRNA purification kit (Ambion, Foster City, CA, USA), and mRNA libraries were constructed using the KAPA RNA HyperPrep kit (Roche, Basel, Switzerland) following the provided procedure. The libraries were sequenced with Illumina Hiseq 4000 (50-bp single-end reads) and analyzed at the Vincent J. Coates Genomics Sequencing Laboratory at UC Berkeley. For preprocessing and demultiplexing of sequencing data, raw sequencing reads were processed using Python scripts (https://github.com/Comai-Lab/allprep/blob/master/allprep-13.py). The mRNA-seq reads were aligned to the reference coding sequences (CDS) of *Diospyros kaki* cv. Taishu whole-genome sequences (Horiuchi et al. 2022) using the Burrows-Wheeler Aligner (version 0.7.15) (Li and Durbin 2009) (http://bio-bwa.sourceforge.net/) and counted per CDS using an R script to calculate the RPKM for each gene, according to Akagi et al. (2014). To understand the reactions related to the predicted rapid-softening fruit, DEGs were detected between the featured areas of the predicted rapid-softening and control fruits in criteria (i) (or RS-F vs C-F), with DESeq2 (Love et al. 2014). To understand the physiological pathways related to the premonitory symptoms of the predicted disordered fruits, DEGs were detected between the featured and non-featured areas in the predicted rapid over-softening fruits in criteria (ii) (or RS-F vs RS-NF), by using edgeR (Robinson et al. 2010, McCarthy et al. 2012), with a paired-test option. Since RS-F and RS-NF have been sampled in each fruit, we adopted edgeR with a paired-test option to detect statistical significance for the comparison of paired samples. Putative functions of each gene were annotated with a BLASTX search of the TAIR10 database (https://www.arabidopsis.org/index.jsp). GO enrichment analysis was performed on the DEGs with shinyGO (http://bioinformatics.sdstate.edu/go/). The threshold for the significance of enriched GO terms was set at *P* < 0.05.

## Supplementary Material

pcad050_Supp

## Data Availability

All Illumina transcriptome sequencing data have been deposited in the DDBJ database: Short Read Archives (SRA) database (BioProject accession number: PRJDB15165, SRA submission ID number: DRA015556 and Run ID numbers: DRR438138–DRR438161).
