# Supplementary material for: Transcriptomic Interpretation on Explainable AI-Guided Intuition Uncovers Premonitory Reactions of Disordering Fate in Persimmon Fruit
Source: Plant Cell Physiol. 2023 May 24;64(11):1323–30. doi: 10.1093/pcp/pcad050 (PMC10700010; doi:10.1093/pcp/pcad050)
Supplement: pcad050_Supp [file pcad050_supp.zip › suppl_data/pcp-2023-e-00010-File007.pdf]

Supplementary materials

Transcriptomic interpretation on explainable AI-guided intuition uncovers premonitory reactions of disordering fate in persimmon fruit

Kanae Masuda[1,*], Eriko Kuwada[1,*], Maria Suzuki[1], Tetsuya Suzuki[2], Takeshi Niikawa[2], Seiichi Uchida[3], Takashi Akagi[1,4,**]

[1] Graduate School of Environmental and Life Science, Okayama University, Okayama, Japan

[2] Faculty of Information Science and Electrical Engineering, Kyusyu University, Fukuoka 819-0395, Japan

[3] Gifu Prefectural Agricultural Technology Center, Gifu, Japan

[4] Japan Science and Technology Agency (JST), PRESTO, Kawaguchi-shi, Saitama 332-0012, Japan

*Contributed equally

**Corresponding author

Email: takashia@okayama-u.ac.jp, TEL: +81-86-251-8337

Supplementary Figure S1

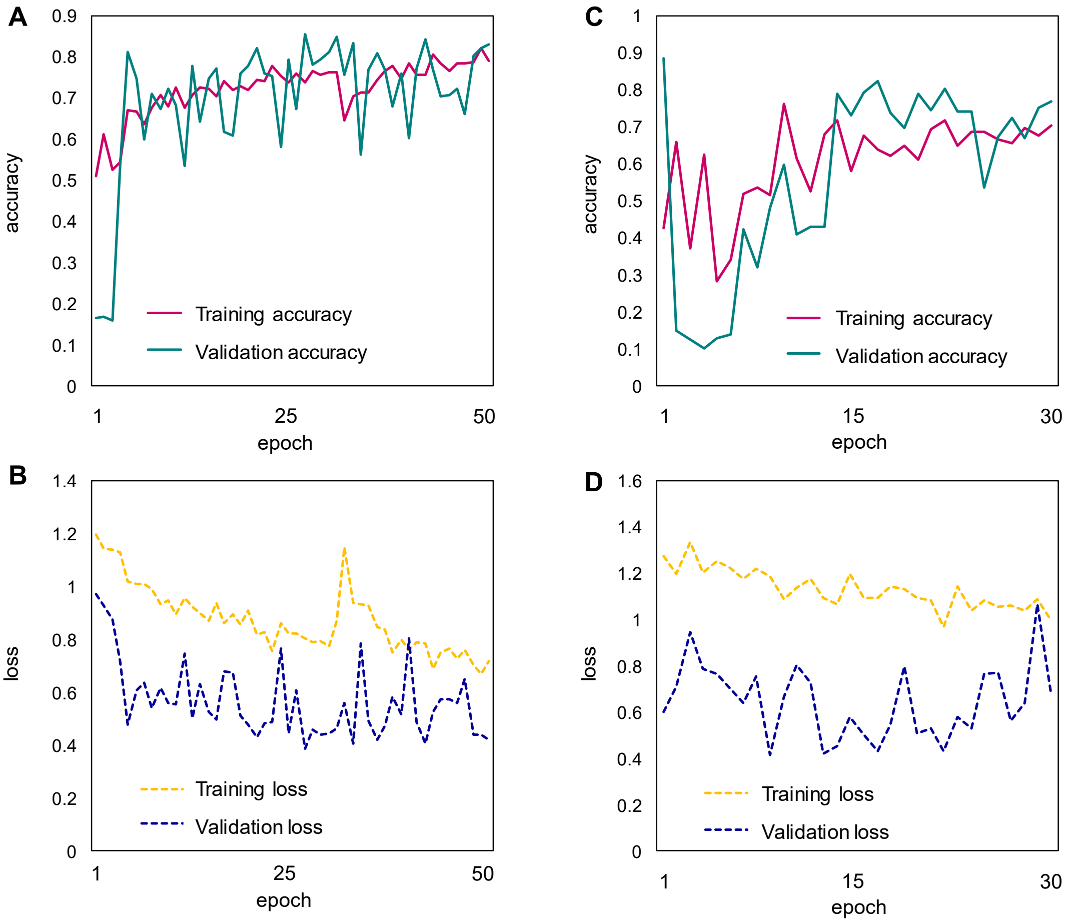

Supplementary Figure S1 Training curves of models A and B for classification of rapid softening persimmon.

Classification accuracy (**A** and **C**) and loss values (**B** and **D**) were given for the training and validation samples, in each epoch. **A-B.** for model A, and **C-D.** for model B.

Supplementary Figure S2

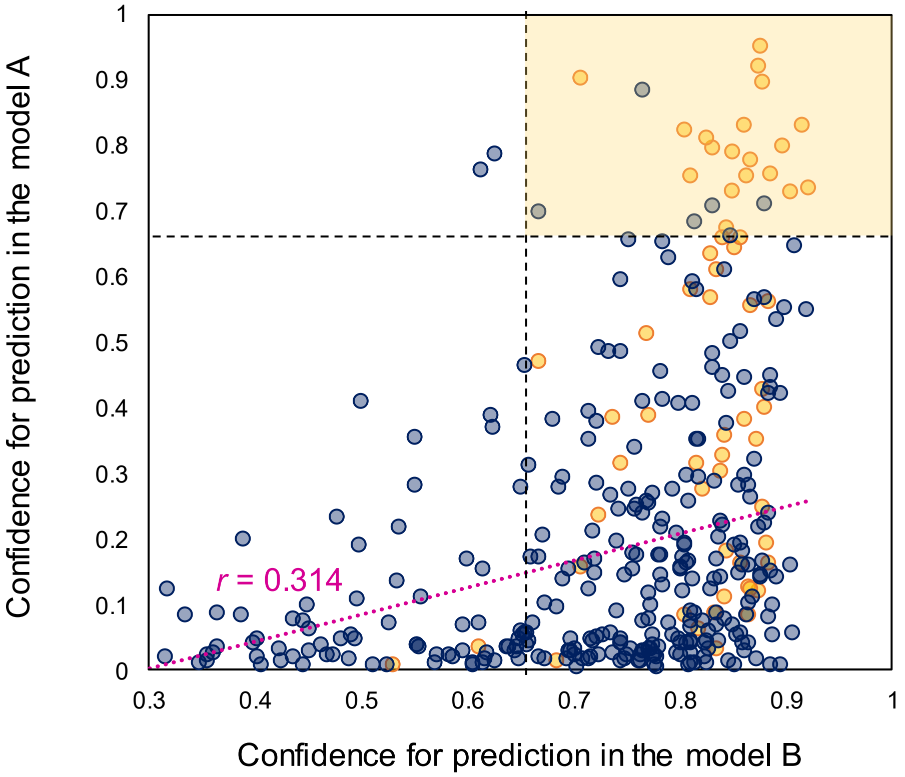

Supplementary Figure S2 Distribution of the confidence for prediction of rapid softening fruits in models A and B, for the common testing samples.

For the validation samples in Dataset A (N = 361), models A and B exhibited not high correlation (r = 0.314). This situation suggested that models A and B captured independent feature characteristics. Although positive precisions of each model were quite low (0.47 and 0.25, respectively), integration of the two models filtering with a higher confidence threshold (= 0.65) than the default one (= 0.5), achieved accuracy > 93%, and the positive precision > 0.8 as given in the pale orange area. Circles in orange and blue represent observed rapid-softening and control fruits, respectively.

Supplementary Figure S3

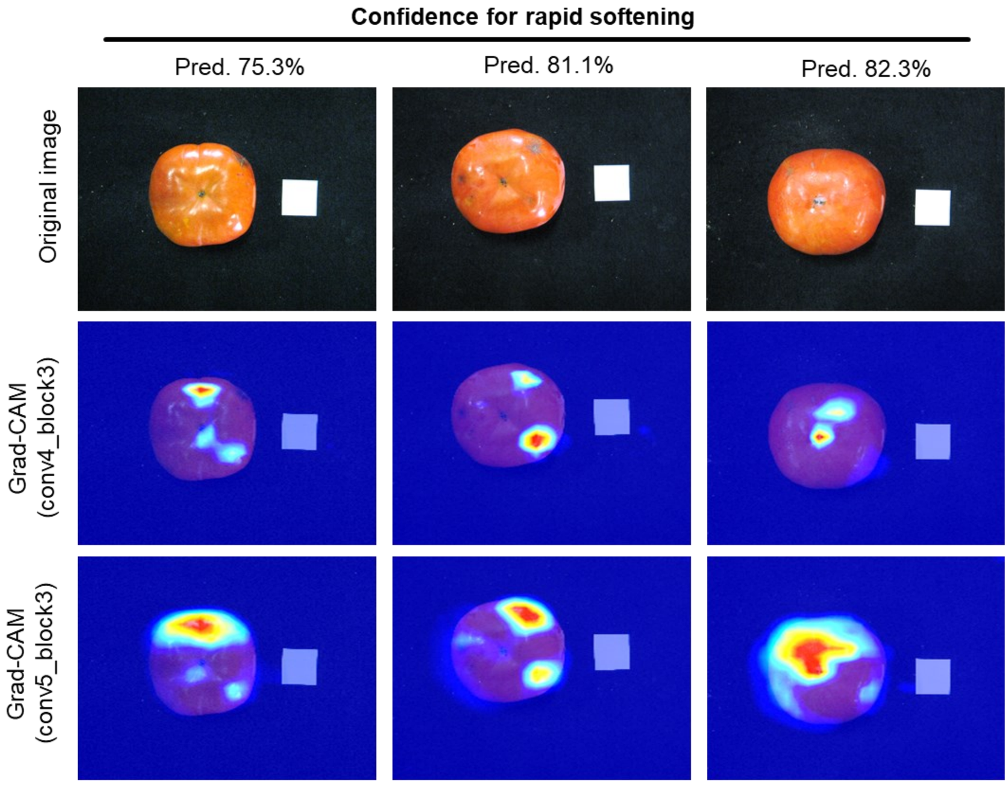

Supplementary Figure S3 Grad-CAM relevance visualization by different convolutional layers. Grad-CAM often gives too coarse relevance visualizations in the feature map by the last convolutional layer (conv5_block3 in VGG16). To show smaller relevance regions, we applied a shallower convolutional layer, conv4_block3 in VGG16. The relevance regions were mostly consistent between the conv4_block3 and conv5_block3 layers, while the conv4_block3 exhibited smaller regions.

Supplementary Figure S4

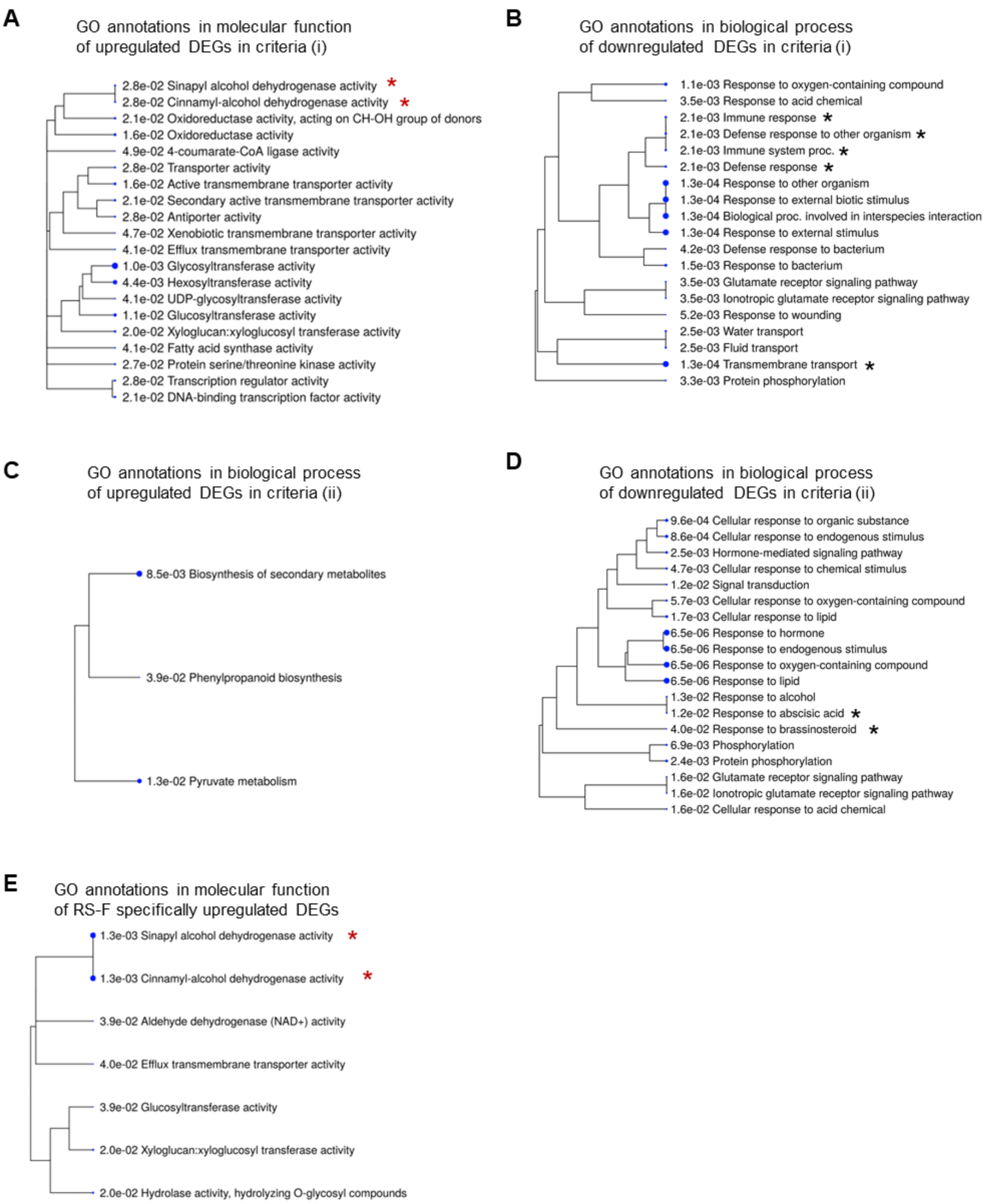

Supplementary Figure S4 GO enrichment analyses for DEGs in criteria (i) and (ii). **A-B**, GO annotations in molecular function and biological process of up- and down-regulated DEGs in criteria (i), respectively. **C-D**, GO annotations in biological process of up- and down-regulated DEGs in criteria (ii). **E**, GO annotations in molecular function of RS-F specifically upregulated DEGs. Larger bule dots indicate more significant FDR values. Asterisks represent GO annotations mentioned in the main text.

Supplementary Table 1 List of the differentially expressed genes in the criteria (i).

Supplementary Table 2 List of the differentially expressed genes in the criteria (ii).

Supplementary Table 3 List of the commonly upregulated and downregulated genes in criteria (i) and (ii).

Supplementary Figure S4 GO enrichment analyses for DEGs in criteria (i) and (ii).

Refer to merged excel files.
